# A molecular analysis of sand fly blood meals in a visceral leishmaniasis endemic region of northwestern Ethiopia reveals a complex host-vector system

**DOI:** 10.1016/j.heliyon.2019.e02132

**Published:** 2019-07-26

**Authors:** Solomon Yared, Araya Gebresilassie, Ibrahim Abbasi, Essayas Aklilu, Oscar D. Kirstein, Meshesha Balkew, Adam S. Brown, Ronald M. Clouse, Alon Warburg, Asrat Hailu, Teshome Gebre-Michael

**Affiliations:** a Department of Biology, Jigjiga University, Jigjiga, Ethiopia; b Department of Zoological Sciences, Addis Ababa University, Addis Ababa, Ethiopia; c The Kuvin Center for the Study of Infectious and Tropical Diseases, Institute for Medical Research, The Hebrew University, Israel; d Department of Biology, Mada Walabu University, Bale-Robe, Ethiopia; e President's Malaria Initiative (PMI) Vector Link Project, Ethiopia; f Harvard University, Department of Biomedical Informatics, Boston, MA, United States; g American Museum of Natural History, New York, NY, United States; h Department of Microbiology, Immunology & Parasitology, School of Medicine; Addis Ababa University, Addis Ababa, Ethiopia; i Aklilu Lemma Institute of Pathobiology, Addis Ababa University, Addis Ababa, Ethiopia

**Keywords:** Agriculture, Ecology, Molecular biology, Zoology

## Abstract

**Background:**

Visceral leishmaniasis (VL, or “kala-azar”) is a major cause of disability and death, especially in East Africa. Its vectors, sand flies (Diptera: Psychodidae: Phlebotominae), are poorly controlled and guarded against in these regions, owing in part to a lack of understanding about their feeding behavior.

**Methods:**

A total of 746 freshly fed female sand flies were collected in five population centers in Kafta Humera (northwestern Ethiopia), where VL is endemic. Flies were collected from habitats that ranged from inside houses to open fields, using light traps and sticky traps. Sources of sand fly blood meals were identified using enzyme-linked immunosorbent assays (ELISA) and DNA amplification with reverse-line blot analysis (PCR-RLB); 632 specimens were screened using ELISA, 408 of which had identifiable blood meals, and 114 were screened using PCR-RLB, 53 of which yielded identifications. Fly species determinations were based on morphology, and those specimens subjected to PCR-RLB were also screened for *Leishmania* parasites using conventional PCR to amplify the nuclear marker ITS1 (internal transcribed spacer 1) with *Leishmania*-specific primers.

**Results:**

More than three-fourths of all sand flies collected were *Phlebotomus orientalis*, and the remaining portion was comprised of nine other species. Nearly two-thirds of *P. orientalis* specimens were collected at village peripheries. The most common blood source for all flies was donkey (33.9% of all identifications), followed by cow (24.2%), human (17.6%), dog (11.8%), and goat or sheep (8.6%); mixtures of blood meals from different sources were found in 28.2% of all flies screened. Unidentified blood meals, presumably from wildlife, not domestic animals, were significantly higher in farm fields. *Leishmania* parasites were not detected in any of the 114 flies screened, not surprising given an expected infection rate of 1–5 out of 1,000. Meals that included a mixture of human and cow blood were significantly more frequent relative to all cow meals than human blood meals were to non-cow meals, suggesting a zoopotentiative interaction between cows and humans in this system.

**Conclusions:**

Habitat and host preferences of sand fly vectors in Kafta Humera confirmed the finding of previous reports that the main vector in the region, *Phlebotomus orientalis*, is a highly opportunistic feeder that prefers large animals and is most commonly found at village peripheries. These results were similar to those of a previous study conducted in a nearby region (Tahtay Adiabo), except for the role of cattle on the prevalence of human blood meals. Preliminary examinations of blood meal data from different settings point to the need for additional surveys and field experiments to understand the role of livestock on biting risks.

## 1 Introduction

Vector control has been shown to be effective against sand fly-borne diseases (WHO, 2010), but no such program has been attempted in northwestern Ethiopia due to the limited historical data on sand fly feeding behavior, reservoir hosts of the parasites they carry, and interactions between sand flies and humans. Sand flies (Diptera: Psychodidae: Phlebotominae) are best known for carrying the *Leishmania* protists that cause visceral leishmaniasis (VL, also commonly known as “kala-azar”) or cutaneous leishmaniasis (CL), as well as other pathogenic bacteria and viruses. Female sand flies feed on vertebrate blood to obtain nutrition for egg development, and, with specificity that varies between different sand fly species, they target a wide variety of hosts, including livestock, dogs, chickens, rodents and reptiles (Haouas et al., 2007; Lainson and Rangel, 2005; WHO, 2010).

Visceral leishmaniasis can cause death if untreated and is caused by the *L. donovani* species complex. However, sand fly vector species for *L. donovani*, the preferred vertebrate for vector blood meals, and the reservoir species for the parasite are more difficult to determine and can vary between regions. *Phlebotomus orientalis* carries *L. donovani* in Eastern Sudan (Elnaiem et al., 1998) and most likely the surrounding regions, including the study sites here. PCR-based and parasitological approaches have identified dogs as an important reservoir of *L. donovani* in Sudan (Dereure et al., 2003; Hassan et al., 2009), and dog ownership has been identified as a risk factor for *L. donovani* infections in humans in Ethiopia (Argaw et al., 2013; Bashaye et al., 2009; Yared et al., 2014). The presence of antibodies show that all common domestic animals in Ethiopia are routinely exposed to sand flies and *L. donovani* (Kenubih et al., 2015; Rohousova et al., 2015), and in northwestern Ethiopia *L. donovani* antibodies were detected more frequently in cattle (41.9%) than all other animals tested (Kenubih et al., 2015). In eastern Sudan this figure for cattle is 21.4% (Mukhtar et al., 2000). However, tests for *Leishmania* DNA in cattle (Alam et al., 2011) have been negative, and it seems likely that cattle are not actually a reservoir for the parasite.

Still, cattle and other livestock may inhibit or drive human exposure to *L. donovani*. Some studies have provided evidence that proximity to animals, and especially cattle, is preventative for sand fly feeding by drawing the flies away from humans (zooprophylaxis) (Bern et al., 2010; Gebre-Michael et al., 2010; Torr et al., 2007), while others argue that animals play no role in exposure (Gebresilassie et al., 2015a, 2015c), reduce sand fly bites by too little to significantly reduce disease risks (Tirados et al., 2011), or actually increase the odds of being bitten by attracting flies to places of human activity (zoopotentiation) (Bern et al., 2010; Yared et al., 2014). Further complicating the debate are observed group size dynamics, which modulate the risk of sand fly encounters (Hebblewhite and Pletscher, 2002), as well as species diversity of host groups, and vector preferences for different hosts (Miller and Huppert, 2013). In addition, computer simulations of malaria vector dynamics have led to the conclusion that vector mortality risk while searching for meals, as determined by the proximity of hosts to mosquito breeding sites, is a critical determinant of zooprophylaxis or zoopotentiation (Saul, 2003).

Investigations into the host preferences and feeding patterns of sand flies under natural conditions are essential to understand their vectorial capacity and to clarify natural transmission cycles (Killick-Kendrick, 1999). Combined with studies of risk factors for contracting the disease (Bern et al., 2010), host choice data can reveal important factors to control so as to lessen exposure to sand flies and subsequent transmission of leishmaniasis. Host choice behavior can depend on biotic factors, such as host size, abundance, and behavior, as well as abiotic factors, such as temperature, wind, heat, humidity, sound, and CO_2_ levels (Bell, 1990; Foster et al., 1972; Gibson and Torr, 1999; Killick-Kendrick, 1999), and sand fly host preferences have been determined by observing the behavior of sand flies presented with different animal baits and by examining the blood meal sources of field-caught specimens (Alexander, 2000; Karaku at al., 2017; Montoya-Lerma and Lane, 1996). The purpose of this study was to use blood meal identification to add to our growing body of knowledge about sand fly feeding behavior in northwestern Ethiopia (Gebre-Michael et al., 2010; Gebresilassie et al., 2015a, 2015c; Kenubih et al., 2015; Rohousova et al., 2015; Yared et al., 2014), connecting domestic animal host identities to vector species and habitats. This would paint a clearer picture of the dominant players in the system and allow us to further characterize the ecology of leishmaniasis in this critical region. Furthermore, we sought to explore our data statistically and compare it to a previous study from a nearby region for more insight into questions of zooprophylaxis and zoopotentiation.

## 2 Material and methods

### 2.1 Study area

This study was conducted in the Ethiopian district of Kafta Humera, which covers over 4,500 km^2^ in the Western Tigray zone, located along the borders of Eritrea and Sudan. In the 2007 census Kafta Humera had a total population of 92,144, with a density of 20.3 persons per km^2^, 67.2% of which was rural (Office of the Census Commission and the Central Statistical Agency of Ethiopia, 2008). The district capital, Humera town, and four villages within a 32-km radius—Rawyan, Mykadra, Bereket, and Adebay—were sampled. The villages are populated by settlers who returned from Sudan in 1993 and 1994, as well as internal settlers originating from different parts of the Tigray region. Residents live in houses of different construction (concrete-or mud-walled, with corrugated iron or thatched roofs) and practice mixed farming (growing crops and engaging in small-scale livestock production). Residents usually keep domestic animals, such as chickens, goats, sheep, donkeys, cattle, and dogs, close to their houses. Field work in the district is performed annually by hundreds of thousands of migrant laborers from different parts of Ethiopia, especially from May to December.

### 2.2 Collection and identification of sand flies

At each of the five study villages, four sand fly collection sites were selected: inside houses, within villages, at the periphery of villages, and in open fields adjacent to villages. Sand fly collections were made two or three nights per month from May 2011 to April 2013. All five localities were sampled through April 2012, which indicated that the density of *P. orientalis* was highest in Adebay; consequently, collection efforts were focused there from December 2012 to April 2013. Sand flies were captured using CDC miniature light traps (John W. Hock Company, Gainesville, FL, USA) and sticky traps (Alexander, 2000). In and around villages traps were deployed close to animal shelters and houses, and in open fields traps were placed close to the ground.

For the collection of sand flies from indoors and from inside and around villages, including farm fields, oral informed consent was obtained from heads of households. Letters of support were also obtained from the Western Tigray and Kafta Humera district health bureaus.

Freshly fed female sand flies could be identified by the blood visible in their abdomens, and they were immediately preserved in alcohol or silica gel grains. In the laboratory, the head and abdominal tips of female sand flies from light traps and sticky traps were removed and mounted on a slide using a drop of Hoyer's medium for species identification. Species were identified based upon the morphology of the cibaria, pharyngeal armature, and spermatheca, using morphological keys and other published materials (Abonnenc and Minter, 1965; Gebre-Michael and Medhin, 1997; Lewis, 1982; Lane and Fritz, 1986; Quate, 1964).

### 2.3 Blood meal analysis

Sand fly blood meals were identified using either enzyme-linked immunosorbent assay (ELISA) or polymerase chain reaction followed by reverse line blot analysis (PCR-RLB). We relied on ELISA to identify blood meals from most of our captured female sand flies (n = 632), but to advance the use of DNA-based methods in leishmaniasis studies in Ethiopia, we used PCR-RLB for 114 specimens. Both methods could identify meals as human, bovine, donkey, goat, or sheep, but only ELISA could identify dog, and only PCR-RLB could identify chicken, mouse, and camel.

While analyses of sand fly blood meals are typically serological (Afonso et al., 2005; Baum et al., 2013; Beier et al., 1988; Burniston et al., 2010; Colmenares et al., 1995; Gebre-Michael et al., 2010; Morsy et al., 1993; Nery et al., 2004; Srinivasan and Panicker, 1992), these tests are time consuming and sometimes lead to misidentification due to cross-reactivity of serum proteins (Ngo and Kramer, 2003). DNA-based techniques have thus gained popularity (Garlapati et al., 2012; Lutomiah et al., 2014; Muturi et al., 2011), including restriction fragment length polymorphism combined with polymerase chain reaction (RFLP-PCR) and Sanger sequencing of the mtDNA genes cytochrome *c* oxidase 1 (COI) and cytochrome *b* (cytb). DNA-based techniques have been used previously to identify blood meal sources in tsetse flies (Muturi et al., 2011), mosquitoes (Lutomiah et al., 2014), ticks (Kirstein and Gray, 1996), and sand flies (Soares et al., 2014; Oshaghi et al., 2009; Valinsky et al., 2014). However, such techniques can require more PCR product than can be generated from samples, and they can fail to detect multiple blood meal sources within a single fly. Recently, the amplification of the mitochondrial gene cytochrome *b*, followed by reverse line blot analysis (PCR-RLB), has been used to identify the origin of blood in ticks (Humair et al., 2007; Scott et al., 2012) and sand flies (Abbasi et al., 2009; Garlapati et al., 2012), and this technique allows the identification of multiple hosts (Abbasi et al., 2009; Garlapati et al., 2012; Scott et al., 2012); thus, our use of PCR-RLB here.

ELISA procedures were performed as described by Beier et al. (1988), Colmenares et al. (1995), and Burniston et al. (2010), with the following specifications for this study. Based on checkerboard titrations, the optimal dilutions of the conjugated antibodies were 1:2,000 μl for anti-human immunoglobulin G (IgG), 1:250 μl for anti-bovine IgG, 1:5,000 μl for anti-donkey and anti-dog IgG, and 1:10,000 μl for anti-goat and anti-sheep IgG (a high dilution to prevent cross-reactions with other species). Negative controls were used from a laboratory colony of unfed females of *P. orientalis*, and positive controls were blood samples known to originate from each of the target species. Plates were read visually and using an ELISA plate reader at 405 nm absorbance, and samples were considered positive if the absorbance (optical density) value was more than three standard deviations higher than the four negative controls.

PCR methods followed those of Abbasi et al. (2009), with the following specifications. DNA was extracted individually from sand fly specimens by digestion in a total volume of 200 μL of lysis buffer, consisting of 50 mM NaCl, 10 mM EDTA, 50 mM Tris–HCl (pH 7.4), 1% triton X-100, and 200 μg/mL of proteinase K. This was followed by extraction with phenol-chloroform and precipitation using ethanol. The precipitated DNA was suspended in Tris-EDTA (TE, 10 mM Tris–HCl [pH 7.4], and 1 mM EDTA) buffer at a concentration of 50 μL. A 344 bp sequence of the conserved region of cytochrome *b* was amplified using the forward primer Cyto1 (5′- CCA TCA AAC ATC TCA GCA TGA TGA AA -3′) and the reverse primer Cyto2 (5′- CCC CTC AGA ATG ATA TTT GTC CTC -3′). The cytochrome *b* region was amplified in a total reaction volume of 50 μL: 0.5 μL of each primer, 5 μL of genomic DNA, 19 μl water, and of 25 μL of HotStarTaq Master Mix (Qiagen, Valencia CA), which consists of 1.5 mM MgCl_2_, 200 μM of each dNTP, 75 mM KCl, and 10 mM Tris-HCl (pH 8.8). The PCR temperature profile was as follows: starting at 95 °C for 5 min; continuing with 35 cycles of 94 °C for 30 s, 55 °C for 30 s, and 72 °C for 1 min; and concluding with 72 °C for 10 min. Cow blood was used as a positive control, and sterile water was the negative control.

Amplified PCR products were then used as probes in RLB hybridization reactions, followed by chromogenic detection, using methods described in Abbasi et al. (2009). The method relies on 5′-amino-linked oligonucleotide probes specific to each host species, designed using publicly available alignments of their cytochrome *b* sequences. The probes were then linked to nylon membranes to which denatured and biotinylated PCR products (if present) were allowed to hybridize. Hybridized DNA was first detected by incubating membranes in streptavidin horseradish peroxidase (HRP), and for chromogenic detection, enhanced chemiluminescence (ECL) detection was performed using the EZ-ECL detection kit (Biological Industries, Beit Haemek, Israel).

Samples used for identification of blood meals by PCR-RLB were also screened for *Leishmania* parasites using conventional PCR, following the methods of Gebresilassie et al. (2015b). The nuclear marker ITS1 (internal transcribed spacer 1) was amplified using the *Leishmania*-specific primers LITSR (5′- CTG GAT CAT TTT CCG ATG -3′) and L5.8S (5′- TGA TAC CAC TTA TCG CAC TT -3′) and then checked by visualization on a 1.5% agarose gel containing ethidium bromide.

## 3 Calculation

To further refine our understanding of sand fly feeding behavior, we performed two statistical explorations of our blood-meal data. First, ratios of blood meals identified as human, animal-fed, and human plus animal were compared between villages and farm fields using Z-tests. Next, binomial logistic regression was performed to measure the odds of identifying human blood meals in conjunction with various factors, as well as to compare it with a previously published data set from Tahtay Adiyabo, also in northwestern Ethiopia (Gebresilassie et al., 2015a). Factors of interest were domestic animals (all combined); cows, donkeys, and dogs (identified only with ELISA in Tahtay Adiyabo); habitat (as a second variable in all tests); and sand fly species (only for Kafta Humera, where multiple species were tested).

Statistical tests were performed in (R Development Core Team, 2012 after combining certain categories of data in the Kafta Humera data set, namely village (Adebay, Bereket, *etc.*), habitat (“inside,” “in village,” and “around village” were combined), and molecular method (ELISA or PCR-RLB). Sand fly and livestock species were also combined except where otherwise noted. The R script used is available in the Supplementary material (S1 Script - Statistical Modeling.R) and at GitHub (github.com/adam-sam-brown/Yared_et al.).

## 4 Results

### 4.1 Sand fly blood meals in Kafta Humera

**Fig. 1:**
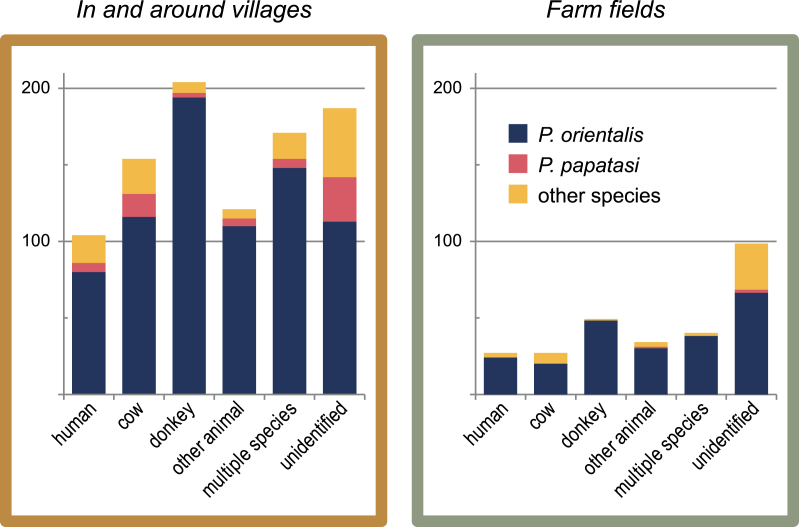
The number of blood meals in Kafta Humera, broken down by habitat, sand fly species, and a selection of blood meal descriptions. Meals identified as originating from specific animals may have been found in combination with other animal or human meals; the number of combined meals is also shown. The most common blood meal identification was donkey, and *P. orientalis* was the most commonly caught sand fly species. Unidentified meals were more common than identified meals in sand fly species other than *P. orientalis*.

**Fig. 2:**
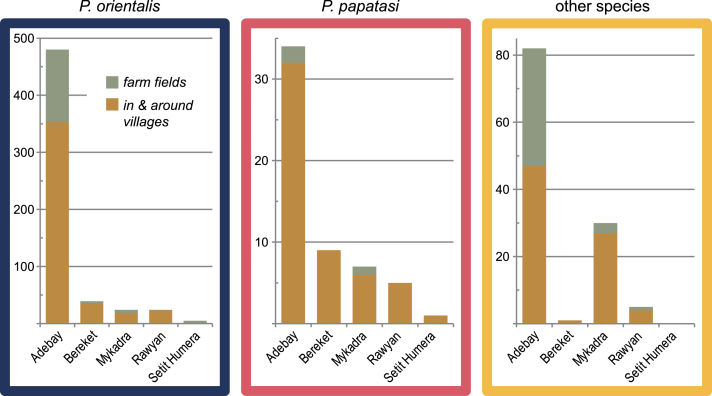
The number of blood meal samples in Kafta Humera district, broken down by sand fly species, simplified binomial habitat category (in and around villages versus in farm fields), and locality. The Adebay locality produced the most specimens, prompting the study to focus efforts there. Most sand fly species caught were identified as *P. orientalis*, and they were most frequent in and around villages.

**Fig. 3:**
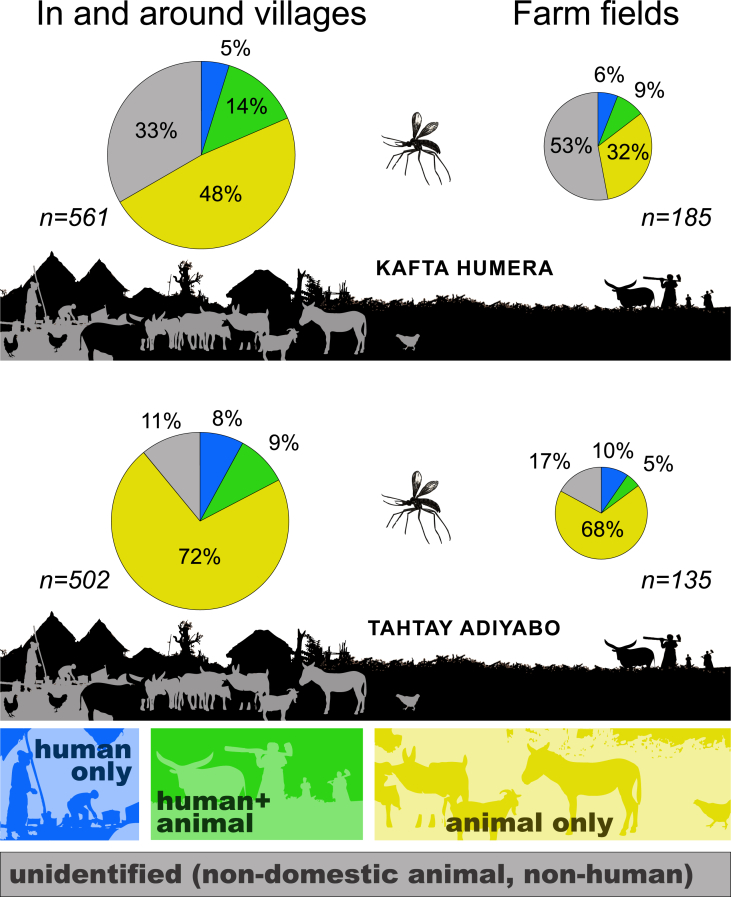
Blood meals represented by habitat and source in Kafta Humera and Tahtay Adiyabo districts. Pie chart areas are proportional to sample sizes and demonstrate the larger proportion of non-human and non-animal blood meals in farm fields, the larger proportion of animal meals in Tahtay Adiyabo, and the larger proportion of combined human and animal meals in Kafta Humera.

In Kafta Humera a total of 746 freshly fed, female sand flies were collected, and from these 461 blood meals were identified (408 by ELISA and 53 by PCR-RLB; S1 Table - Kafta Humera data.csv). Most specimens collected were *P. orientalis* (76.1%; Fig. 1), from the village of Adebay (80.1%; Fig. 2), and collected at village peripheries (62.3%). The other sand fly species collected were, in order of abundance, *Sergentomyia clydei* (8.4%), *Phlebotomus papatasi* (7.5%), *Sergentomyia schwetzi* (4.6%), *Phlebotomus bergeroti* (1.5%), *Sergentomyia africana* (0.8%), *Sergentomyia bedfordi* (0.4%), *Phlebotomus alexandri* (0.2%), *Phlebotomus duboscqi* (0.2%), and *Phlebotomus lesleyae* (0.1%). Human blood was identified in 17.6% of the blood meals, donkey blood in 33.9%, cow blood in 24.2%, dog blood in 11.8%, and goat or sheep blood in 8.6% (results of goat and sheep blood meals were combined due to cross reactions between these species); blood meals from 210 sand flies (28.2% of the total collection) were from multiple sources (see Fig. 3).

Among identified blood meals from *P. orientalis* (*n* = 389), *P. papatasi* (25), other *Phlebotomus* species (7), and *Sergentomyia* (40), each group had human and livestock meals, feedings comprised of multiple species, and feedings from livestock and humans in farm fields, village peripheries, and inside villages and homes. However, *P. orientalis* appeared to prefer donkeys over cattle (found in 62% *vs.* 35% of identified meals, respectively), in contrast with *P. papatasi* (12% *vs.* 60%) and *Sergentomyia* species (17% *vs.* 66%). Multiple host species were identified in 48% of known *P. orientalis* blood meals but only 20% of those from *P. papatasi*. Also, although 25% of *P. orientalis* specimens were captured in farm fields and 7% in villages (including indoors), those figures were 5% and 39% for *P. papatasi*, respectively (village peripheries constituting the remaining captures).

Chicken, mouse, and camel meals were not common: two specimens had chicken blood (combined with cow and mouse), three were from mouse (two combined with cow and one with chicken), and four were from camels (two combined with cow).

*Leishmania* parasites were not detected in any of the 114 samples screened by PCR-RLB and checked for the parasite using conventional PCR to amplify the *Leishmania* ITS1 nuclear region; this was not unexpected, given a reported vector infection rate in northern Ethiopia of 0.1–0.5% by Gebresilassie et al. (2015b).

Blood meals that were unidentified (and presumably from wildlife, for which our methods did not test) were significantly more common in farm fields (p < 0.0001, Z-test; Table 1). In addition, blood meals identified as coming only from domestic animals were significantly lower in farm fields as a proportion of all blood meals (p = 0.0003, Z-test).

**Table 1:** Number of blood meals identified as human, animal, or a combination of both, separated by habitat (in and around villages *vs.* in farm fields), from two different localities in northwestern Ethiopia. Percentages are out of the total number of specimens caught in that region and habitat. P-values compare the ratios of each subset between habitats (<0.10 in bold).

	Villages	Farm fields	p
**Kafta Humera**			
Human	27 (5%)	11 (6%)	0.68
Human + Domestic Animal	77 (14%)	16 (9%)	0.09
Domestic Animal	270 (48%)	60 (32%)	**0.0003**
Unidentified	187 (33%)	98 (53%)	**<0.0001**
Total	561	185	
**Tahtay Adiyabo**			
Human	40 (8%)	13 (10%)	0.66
Human + Domestic Animal	47 (9%)	7 (5%)	0.17
Domestic Animal	360 (72%)	92 (68%)	0.48
Unidentified	55 (11%)	23 (17%)	**0.08**
Total	502	135	

**Fig. 4:**
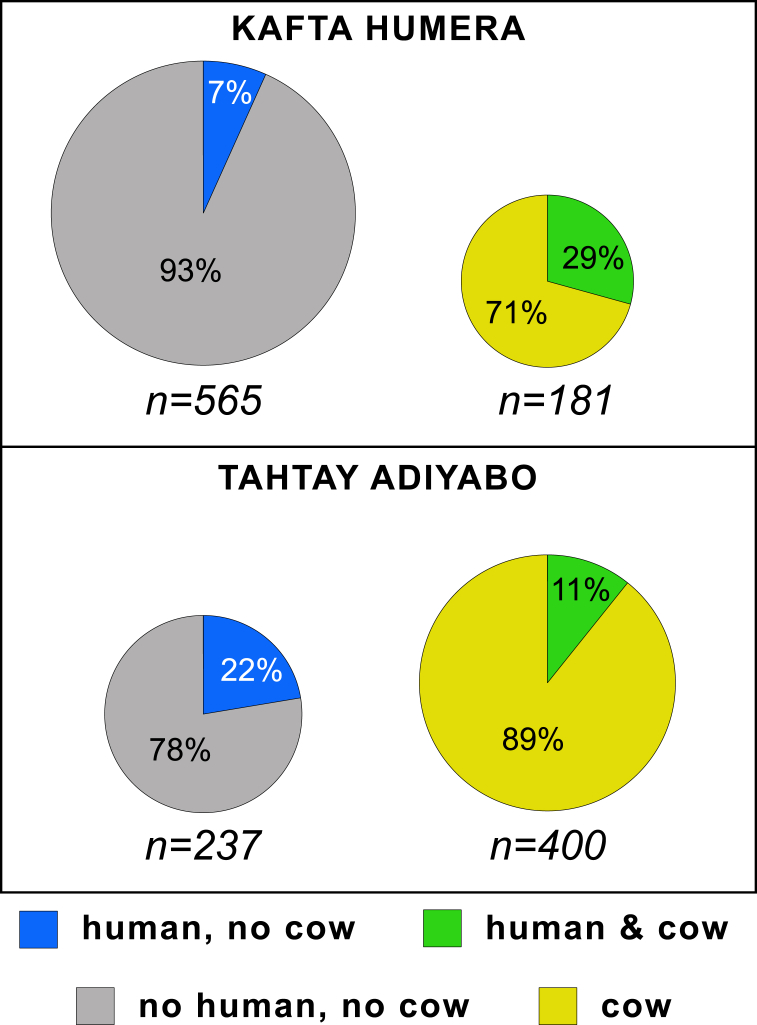
Host overlap in phlebotomine blood meals. Human and cow blood meals, both in combination and separately, for Kafta Humera and Tahtay Adiyabo. Pie chart sizes are in proportion to sample sizes, and these and similar ratios were the basis of the binomial logistic regression analyses.

In addition, the odds of identifying human blood meals more than doubled when they were associated with domestic animal blood meals (p < 0.001). This was primarily due to cow blood meals, which increased the odds of human blood meals 2.54 times (p < 0.0001; Table 2, Fig. 4). Blood meals from other domestic animals, the species of sand fly, and the habitat had no effect on the odds of identifying human blood meals in Kafta Humera.

**Table 2:** Odds radios calculated through binomial logistic regression for factors related to human blood meals: found in combination with domestic animal blood meals (combined or with certain species of interest), and recovered from different habitats (in and around villages versus in farm fields). All fly species were combined except for one test in Kafta Humera, where *P. orientalis vs.* other fly species was coded as a binomial variable (shown).

Variable	Kafta Humera			Tahtay Adiyabo		
	ln OR	Simple OR	p	ln OR	Simple OR	p
Domestic Animal	0.72	2.06	**<0.001**	-1.78	0.17	**<<0.00001**
Habitat	0.14	1.15	0.55	0.39	1.48	0.17
Fly species	-0.03	0.97	0.91			
Cow	0.93	2.54	**<0.0001**	-1.17	0.31	**<0.00001**
Habitat	0.15	1.16	0.53	0.41	1.51	0.14
Donkey	0.24	1.28	0.22	0.87∗	2.39	**0.001**
Habitat	0.26	1.30	0.27	0.66∗,†	1.93	**0.07**
Dog	-0.23	0.79	0.47	-0.27∗	0.76	0.51
Habitat	0.29	1.34	0.22	0.55∗	1.73	0.13

P-values less than 0.10 are in bold.

∗ ELISA data only.

† Simple OR of donkey exposure (village/field) = 1.81, OR of being bitten (village/field) = 2.05, average 1.93.

### 4.2 Comparisons with Tahtay Adiyabo district

Previously published data from Tahtay Adiyabo (Gebresilassie et al., 2015a) are briefly described again here (S2 Table - Tahtay Adiyabo data.csv). From 637 freshly fed female *P. orientalis* 559 blood meals were identified (422 by ELISA and 137 by PCR-RLB). Human blood was identified in 8.3% of all blood meals, donkey blood in 24.7% (using ELISA only), cow blood in 62.8%, dog blood in 16.4% (using ELISA only), and goat or sheep blood in 6.0%; blood meals from 54 sand flies (8.5% of the total collection) were from multiple sources (see Fig. 3).

In contrast to results from Kafta Humera obtained here, in Tahtay Adiyabo (Gebresilassie et al., 2015a) identification of human blood meals decreased by 83% when found in combination with domestic animals (p < 0.00001; Table 2, Fig. 4). This effect was again primarily due cattle blood meals, with the odds of human blood meals being 69% lower in combination with those from cows (p < 0.00001). Identifying human blood meals was 2.39 more likely in combination with donkey blood meals in Tahtay Adiyabo (p = 0.001), and there was a trend for a doubling of the odds of human meals in conjunction with donkeys when in and around villages versus in farm fields (but this was not significant, p = 0.07). Blood meals from dogs showed no influence on the odds of identifying human blood meals in Tahtay Adiyabo.

## 5 Discussion

### 5.1 Feeding trends

Consistent with the findings of Quate (1964) and Gebre-Michael et al. (2010), we found that *P. orientalis* is found predominantly at the periphery of villages and in farm fields, and, consistent with Yared et al. (2017) and Gebre-Michael et al. (2010), we found it to be an opportunistic feeder, having blood meals from every species targeted by our methods. We also found that *P. papatasi*, like *P. orientalis*, is an opportunistic feeder (including on chickens), and this is consistent with studies in Turkey (Svobodova et al., 2003), Israel (Lutomiah et al., 2014), Italy (Bongiorno et al., 2003), Yugoslavia (Kostich, 1951), Egypt (el Sawaf et al., 1989), and Iran (Javadian et al., 1977; Yaghoubi-Ershadi et al., 1995). In Nepal *P. papatasi* was shown to be highly anthropophilic (Burniston et al., 2010), and in many regions of the Old World *P. papatasi* is commonly found inside human dwellings. Thus, *P. papatasi* has become an effective vector for CL (WHO, 2010). Nonetheless, mixed blood meals for *P. papatasi* in this study were not as frequent as they were in *P. orientalis* (20% of identified meals *vs.* 48%), and the same has been observed for *P. papatasi* relative to *P. argentipes* (Palit et al., 2005).

At least two *Sergentomyia* species, *S. schwetzi* and *S. clydie*, were also found to be opportunistic feeders in this study, and they frequently fed on humans, often in combination with other hosts; indeed one *S. clydie* specimen was found to have a blood meal consisting of cow, dog, donkey, *and* human blood. The genus *Sergentomyia* comprises species that are highly abundant in Kafta Humera and which occur throughout the year. They are known vectors of the subgenus *Sauroleishmania* (implicated in CL) and have been previously shown to feed on a variety of mammals (including humans) and reptiles in Ethiopia and the surrounding regions (Heisch et al., 1956; Javadian et al., 1977; Ngumbi et al., 1992; Polanska et al., 2014; Quate, 1964; Srinivasan and Panicker, 1992). DNA of *Leishmania major* (the main cause of CL) has been detected in *S. darlingi* in Mali (Berdjane-Brouk et al., 2012), but a study in Ethiopia did not incriminate *S. schwetzi* as a vector of *L. donovani* or other *Leishmania* species (Sadlova et al., 2013).

These and other variations in blood meal composition among different sand fly species may be the result of sand fly preferences, and indeed bait tests of *P. orientalis* conducted in nearby Tahtay Adiyabo district found that cows received the most fly visits, and flies on donkeys had the highest rate of engorgement (Gebresilassie et al., 2015c). Nonetheless, other causes include host availability, density, size, and biomass, as well as trap positioning and trapping efficiency (Bongiorno et al., 2003; Dye et al., 1991; Garlapati et al., 2012; Quinnell et al., 1992).

### 5.2 Donkey, cow, and dog meals

Factors that may influence the large number of meals identified as donkey in our study include their large body size and the frequency of *P. orientalis* in village peripheries and farm fields, where donkeys are commonly found. We observed that donkeys are usually active at night, engaged in different activities, or tied in compounds separate from other animals, a situation that may help *P. orientalis* easily find and access donkeys. Earlier studies of donkeys in the region have found antibodies against *P. orientalis* (Kolářová et al., 2012; Rohousova et al., 2015), and up to a third of them exposed to *L. donovani* (Kenubih et al., 2015; Rohousova et al., 2015). In Sudan a high prevalence of anti-*L. donovani* antibodies in donkeys has also been detected (Mukhtar et al., 2000), and in the New World donkeys seropositive for *Leishmania braziliensis* have been reported (Truppel et al., 2014).

Although less frequently found than donkey blood meals in our study, cows are clearly an important target for sand flies. Our study recovered far fewer cow blood meals in *P. orientalis* in Kafta Humera than were reported from a similar study in the same region (23.9% *vs.* 91.6%) (Gebre-Michael et al., 2010), a difference that could be due to variations in sand fly collection times or the availability and density of cattle in the collection area (Dinesh et al., 2001). The importance of cattle as a blood meal for sand flies has been reported elsewhere: in the Indian subcontinent the vector species *P. argentipes* is five times more frequent on bovine than human bait (Dinesh et al., 2001), cattle blood has frequently been identified in *P. argentipes* (Garlapati et al., 2012), and calculations of the forage ratios for each host species indicated that cows and pigs were the preferred hosts of *Lutzomyia longipalpis* in Colombia (Morrison et al., 1993).

An important consequence of *P. orientalis* feeding preferentially on large livestock (*i.e.*, cattle and donkeys, as opposed to sheep, goats, dogs, and chickens) is that a large biomass of this sand fly species can be maintained via this behavior. By sustaining the life cycle of these leishmaniasis vectors in abundance, the disease can remain endemic in areas where cattle herding is important.

It was unexpected that blood meals from humans showed no significant association with those from dogs, for dogs have been implicated in other studies as major players in the leishmaniasis system, not the least of which is their role as a proven reservoir of *L. donovani* in Sudan (Dereure et al., 2003; Hassan et al., 2009). In northwestern Ethiopia dogs have been shown to have high seropositivity for *P. orientalis* (Rohousova et al., 2015) and *L. donovani* (Kalayou et al., 2011; Kenubih et al., 2015; Rohousova et al., 2015) antigens, and owning dogs is a significant risk factor for VL (Argaw et al., 2013; Bashaye et al., 2009; Yared et al., 2014). Other studies (Macedo-Silva et al., 2014; Maleki-Ravasan et al., 2009) have found dog blood in the vectors *Lu. longipalpis*, *Phlebotomus perniciosus* and *Phlebotomus perfeliewi*, and dogs were found to play an important role in the zoonotic transmission of *Leishmania infantum* in the Old and New World (WHO, 2010). One hypothesis that is consistent with our data and those from previous studies is that the VL risk from dogs derives from their parasite load and probability of infection from vector bites, not higher vector contact.

### 5.3 Zooprophylaxis

Our finding that the odds of identifying human blood meals are significantly different when in combination with cow and donkey blood meals is consistent with the demonstrated attractiveness of cattle and donkeys to sand flies (Gebresilassie et al., 2015c), and important given the high rate of exposure of cattle to *L. donovani* shown in northwestern Ethiopia (Kenubih et al., 2015). Our finding of different odds ratios between Kafta Humera and Tahtay Adiyabo could be caused by a mechanism in which small cattle herds attract sand flies but cannot accommodate their feeding needs, but large cattle herds both attract and dilute sand fly feeding. That is, cattle and donkeys may provide zoopotentiation in moderate numbers, and, at least for cattle, confer zooprophylaxis in large numbers.

Host abundance has been shown to be a critical variable in complex host-parasite or predator-prey outcomes (Hebblewhite and Pletscher, 2002; Miller and Huppert, 2013), but were cattle more abundant in Tahtay Adiyabo—where cattle blood meals were less likely to be associated with human blood meals, suggesting zooprophylaxis—than Kafta Humera? Indeed, cattle density has been estimated to be twice as large Tahtay Adiyabo, at 1001–2000 per 1000 people, than in Kafta Humera (501–1000 per 1000 people, Jabbar at al., 2007). Moreover, we observed in Kafta Humera during our study that a significant portion of the cattle had been taken elsewhere for grazing. The two regions are otherwise demographically quite similar; Tahtay Adiyabo's total population was within one percent of Kafta Humera's in the 2007 census, and having a comparable area, its population density was also very similar (23.5 people per km^2^). Both populations are also mostly rural, especially that of Tahtay Adiabo (91.6% *vs.* 67.2%).

## 6 Conclusion

The Kafta Humera district of northwestern Ethiopia has several opportunistic sand fly species that feed on both humans and domestic animals. The most common species by far is *P. orientalis*, constituting more than three-fourths of all specimens caught, and nearly half of all *P. orientalis* specimens had meals from a mixture of sources. The common sand fly species had more blood meals from cows and/or donkeys than other domestic animals, but in this system (in contrast to nearby Tahtay Adiabo), *P. orientalis* had nearly twice as many meals from donkeys as cattle. Although *P. orientalis* specimens were caught in all habitats, they were more common at village peripheries and farm fields than in and around houses. Human blood was identified in 17.6% of all blood meals from all fly species, and 8.3% of meals from *P. orientalis.*

We also explored statistical associations between human blood meals and domestic animals, vector species, and habitats. As expected, in farm fields unidentified blood meals were more frequent, and domestic animal meals less frequent, than in villages. Blood meals from humans were significantly more frequent when associated with cow blood meals, in contrast with habitat and fly species, which showed no association; previously published data from Tahtay Adiabo showed the opposite effect (a decrease in human blood meals when associated with meals identified as cow). This opens the question of whether herd size or other local factors can change a zooprophylaxis effect to one of zoopotentiation, and it suggests a highly complex vector dynamic in the leishmaniasis transmission ecology of northwestern Ethiopia.

Vector ecology studies in the future in this region should directly measure herd size and species composition, as well as their proximity to at-risk farmers and village residents. As with any host-parasite-vector system, there are several factors and variables involved, so multivariate statistics are important during analysis. Several recent studies have produced contradictory results, so attention should be drawn to similar patterns among as many important variables as possible. Even seemingly settled questions, like the importance of cows in this system, can be called into doubt by findings such as those of Rohousova et al. (2015), also in northwestern Ethiopia, who observed exposure levels to *P. orientalis* and *L. donovani* in cows at only 5% and 1%, respectively, with a sample size of 101. As in other studies, they did find high levels of exposure to both vectors and parasites in dogs (59% and 56%, respectively), but in our study and that of Gebresilassie et al. (2015a) dogs were minor players and showed no evidence of being strongly associated with the frequencies of human blood meals.

Another necessary avenue for future research is the extension of measures of blood meal frequencies to biting risks and disease risks in the same study. If dogs, for example, do not alter biting risks but are more effective hosts of *L. donovani* than cows, then cows may provide zooprophylaxis in any number. Comparing infection rates to vector behavior from different studies may not be productive, as a broad set of variables that differ among localities may, as we have suggested here, cause very different outcomes.

## Declarations

### Author contribution statement

Solomon Yared: Conceived and designed the experiments; Performed the experiments; Analyzed and interpreted the data; Wrote the paper.

Araya Gebresilassie: Performed the experiments; Contributed reagents, materials, analysis tools or data; Wrote the paper.

Ibrahim Abbasi, Essayas Aklilu: Performed the experiments.

Oscar D. Kirstein: Analyzed and interpreted the data.

Meshesha Balkew, Asrat Hailu, Teshome Gebre-Michael: Conceived and designed the experiments; Analyzed and interpreted the data; Wrote the paper.

Adam S. Brown, Ronald M. Clouse: Analyzed and interpreted the data; Wrote the paper.

Alon Warburg: Conceived and designed the experiments; Analyzed and interpreted the data; Contributed reagents, materials, analysis tools or data; Wrote the paper.

### Funding statement

This study was supported by Bill and Melinda Gates Foundation Global Health Program (grant number OPPGH5336).

### Competing interest statement

The authors declare no conflict of interest.

### Additional information

No additional information is available for this paper.

## Appendix A Supplementary data

S1 Table - Kafta Humera data All blood meal data from Kafta Humera (collected in this study), in comma-separated format. S1 Table - Kafta Humera data

S2 Table - Tahtay Adiyabo data All blood meal data from Tahtay Adiyabo, as previously reported in Gebresilassie et al., 2015a, in comma-separated format. S2 Table - Tahtay Adiyabo data

S1 Script - Statistical Modeling.R Scripts used to conduct logistic regression analyses on data in “S1 Table - Kafta Humera data.csv” and “S2 Table - Tahtay Adiyabo data.csv,” easily modified to test effects of particular animals, vector species, and other variables. S1 Script - Statistical Modeling.R

## References

[bib1] Abbasi I., Cunio R., Warburg A. (2009). Identification of blood meals imbibed by phlebotomine sand flies using cytochrome b PCR and reverse line blotting. Vector Borne Zoonotic Dis..

[bib2] Abonnenc E., Minter D.M. (1965). Bilingual key for the identification of Sand flies of the Ethiopian region. Cah. ORSTOM Entomol. Med..

[bib3] Afonso M.M., Gomes A.C., Meneses C.R., Rangel E.F. (2005). Studies on the feeding habits of *Lutzomyia (N.) intermedia* (Diptera, Psychodidae), vector of cutaneous leishmaniasis in Brazil. Cad. Saúde Pública.

[bib4] Alam M.S., Ghosh D., Khan G.M. (2011). Survey of domestic cattle for anti-*Leishmania* antibodies and *Leishmania* DNA in a visceral leishmaniasis endemic area of Bangladesh. BMC Vet. Res..

[bib5] Alexander B. (2000). Sampling methods for phlebotomine sandflies. Med. Vet. Entomol..

[bib6] Argaw D., Mulugeta A., Herrero M., Nombela N., Teklu T., Tefera T., Belew Z., Alvar J., Bern C. (2013). Risk factors for visceral leishmaniasis among residents and migrants in Kafta-Humera, Ethiopia. PLoS Neglected Trop. Dis..

[bib7] Bashaye S., Nombela N., Argaw D., Mulugeta A., Herrero M., Nieto J., Chicharro C., Cañavate C., Aparicio P., Vélez I.D., J., A., Bern C. (2009). Risk factors for visceral leishmaniasis in a new epidemic site in Amhara Region, Ethiopia. Am. J. Trop. Med. Hyg..

[bib8] Baum M., Ribeiro M.C., Lorosa E.S., Damasio G.A., Castro E.A. (2013). Eclectic feeding behavior of *Lutzomyia (Nyssomyia) intermedia* (Diptera, Psychodidae, Phlebotominae) in the transmission area of American cutaneous leishmaniasis, state of Paran , Brazil. Rev. Soc. Bras. Med. Trop..

[bib9] Beier J.C., Perkins P.V., Wirtz R.A., Koros J., Diggs D., Gargan T.P., II, Koech D.K. (1988). Blood meal identification by direct-enzyme linked immunosorbent assay (ELISA), tested on Anopheles (Diptera: Culicidae) in Kenya. J. Med. Entomol..

[bib10] Bell W.J. (1990). Searching behavior patterns in insects. Annu. Rev. Entomol..

[bib11] Berdjane-Brouk Z., Koné A.K., Djimdé A.A., Charrel R.N., Ravel C., Delaunay P., del Giudice P., Diarra A.Z., Doumbo S., Goita S., Thera M.A., Depaquit J., Marty P., Doumbo O.K., Izri A. (2012). First detection of *Leishmania major* DNA in *Sergentomyia (Spelaeomyia) darlingi* from cutaneous leishmaniasis foci in Mali. PLoS One.

[bib12] Bern C., Courtenay O., Alvar J. (2010). Of cattle, sand flies and men: a systematic review of risk factor analyses for South Asian visceral leishmaniasis and implications for elimination. PLoS Neglected Trop. Dis..

[bib13] Bongiorno G., Habluetzel A., Khoury C., Maroli M. (2003). Host preferences of phlebotomine sand flies at a hypoendemic focus of canine leishmaniasis in central Italy. Acta Trop..

[bib14] Burniston I., Roy L., Picado A., Das M., Rija S., Rogers M., Coosemans M., Boelaert M., Davies C., Cameron M. (2010). Development of an enzyme-linked immunosorbent assay to identify host-feeding preferences of *Phlebotomus* species (Diptera: Psychodidae) in endemic foci of visceral leishmaniasis in Nepal. J. Med. Entomol..

[bib16] Colmenares D.M., Portus M., Botet J., Dobano C., Gallego M., Wolff M., Seguí G. (1995). Identification of blood meals of Phlebotomus perniciosus (Diptera: Psychodidae) in Spain by a competitive enzyme-linked immunosorbent assay biotin/avidin method. J. Med. Entomol..

[bib17] Dereure J., El-Safi S.H., Bucheton B., Boni M., Kheir M.M., Davoust B., Pratlong F., Feugier E., Lambert M., Dessein A., Dedet J.P. (2003). Visceral leishmaniasis in eastern Sudan: parasite identification in humans and dogs; host-parasite relationships. Microb. Infect..

[bib18] Dinesh D.S., Ranjan A., Palit A., Kishore K., Kar S.K. (2001). Seasonal and nocturnal landing/biting behaviour of *Phlebotomus argentipes* (Diptera: Psychodidae). Ann. Trop. Med. Parasitol..

[bib19] Dye C., Davies C.R., Lainson R. (1991). Communication among phlebotomine sand flies: a field study of domesticated *Lutzomyia longipalpis* populations in Amazonian Brazil. Anim. Behav..

[bib20] Elnaiem D.A., Hassan K.H., Ward R.D., Miles M.A., Frame I.A. (1998). Infection rates of *Leishmania donovani* in *Phlebotomus orientalis* from visceral leishmaniasis focus Eastern Sudan. Ann. Trop. Med. Parasitol..

[bib21] el Sawaf B.M., Mansour N.S., el Said S.M., Daba S., Youssef F.G., Kenawy M.A., Beier J.C. (1989). Feeding patterns of *Phlebotomus papatasi* and *Phlebotomus langeroni* (Diptera: Psychodidae) in el agamy, Egypt. J. Med. Entomol..

[bib22] Foster A.W., Boreham L.F.P., Tempelis H.C. (1972). Studies on leishmaniasis in Ethiopia IV: feeding behaviour of *Phlebotomus longipes* (Diptera: Psychodidae). Ann. Trop. Med. Parasitol..

[bib23] Garlapati R.B., Abbasi I., Warburg A., Poché D., Poché R. (2012). Identification of bloodmeals in wild caught blood fed *Phlebotomus argentipes* (Diptera: Psychodidae) using cytochrome b PCR and reverse line blotting in Bihar, India. J. Med. Entomol..

[bib27] Gebre-Michael T., Medhin G. (1997). Morphometric separation of the females of *Phlebotomus* (*Phlebotomus) duboscqi* and *P.* (*P.) bergeroti* (Diptera: Psychodidae). J. Med. Entomol..

[bib24] Gebre-Michael T., Balkew M., Berhe N., Hailu A., Mekonnen Y. (2010). Further studies on the phlebotomine sandflies of the kala-azar endemic lowlands of Humera-Metema (north-west Ethiopia) with observations on their natural blood meal sources. Parasites Vectors.

[bib25] Gebresilassie A., Abbasi I., Aklilu E., Yared S., Kirstein O.D., Moncaz A., Warburg A., Hailu A., Gebre-Michael T. (2015). Host-feeding preference of *Phlebotomus orientalis* (Diptera: Psychodidae) in an endemic focus of visceral leishmaniasis in northern Ethiopia. Parasites Vectors.

[bib26] Gebresilassie A., Abbasi I., Kirstein O.D., Aklilu E., Yared S., Tekie H., A., W., A., H., Gebre-Michael T. (2015). Physiological age structure and *Leishmania* spp. detection in *Phlebotomus (Larroussius) orientalis* (Parrot, 1936) (Diptera: Psychodidae) at an endemic focus of visceral leishmaniasis in Northern Ethiopia. J. Trop. Med..

[bib28] Gebresilassie A., Yared S., Aklilu E., Kirstein O.D., Moncaz A., Tekie H., Warburg A., Hailu A., Gebre-Michael T. (2015). Host choice of *Phlebotomus orientalis* (Diptera: Psychodidae) in animal baited experiments: a field study in Tahtay Adiyabo district, northern Ethiopia. Parasites Vectors.

[bib29] Gibson G., Torr S.J. (1999). Visual and olfactory responses of haematophgous Diptera to host stimuli. Med. Vet. Entomol..

[bib30] Haouas N., Pesson B., Boudabous R., Dedet J.P., Babba H., Ravel C. (2007). Development of a molecular tool for the identification of *Leishmania* reservoir hosts by blood meal analysis in the insect vectors. Am. J. Trop. Med. Hyg..

[bib31] Hassan M.M., Osman F.O., El-Raba'a M.F., Schallig D.H., Elnaiem D.A. (2009). Role of the domestic dog as a reservoir host of Leishmania donovani in Eastern Sudan. Parasites Vectors.

[bib32] Hebblewhite M., Pletscher D.H. (2002). Effects of elk group size on predation by wolves. Can. J. Zool..

[bib33] Heisch R.B., Guggisberg C.A.W., Teesdale C. (1956). Studies in leishmaniasis in East Africa. II. The sand flies of Kitui kala-azar area in Kenya, with the description of six new species. Trans. R. Soc. Trop. Med. Hyg..

[bib34] Humair P.-F., Douet V., Cadenas F.M., Schouls L.M., Van De Pol I., Gern L. (2007). Molecular identification of bloodmeal source in Ixodes ricinus ticks using 12S rDNA as a geneticmarker. J. Med. Entomol..

[bib35] Jabbar M., Negassa A., Gidyelew T. (2007). Geographic distribution of cattle and shoats populations and their market supply sheds in Ethiopia.

[bib36] Javadian E., Tesh R., Saidi S., Nadim A. (1977). Studies on the epidemiology of sand fly fever in Iran. III. Host-feeding patterns of *Phlebotomus papatasi* in an endemic area of the disease. Am. J. Trop. Med. Hyg..

[bib37] Kalayou S., Tadelle H., Bsrat A., Abebe N., Haileselassie M., Schallig H.D. (2011). Serological evidence of *Leishmania donovani* infection in apparently healthy dogs using direct agglutination test (DAT) and rk39 dipstick tests in Kafta Humera, north-west Ethiopia. Transboundary Emerg. Dis..

[bib38] Karaku Ş.M., Pekağ Irba Ş.M., Demir S., Eren H., Töz S., Özbel Y. (2017). Molecular screening of *Leishmania* spp. infection and bloodmeals in sandflies from a leishmaniasis focus in southwestern Turkey. Med. Vet. Entomol..

[bib39] Kenubih A., Dagnachew S., Almaw G., Abebe T., Takele Y., Hailu A., Lemma W. (2015). Preliminary survey of domestic animal visceral leishmaniasis and risk factors in north-west Ethiopia. Trop. Med. Int. Health.

[bib40] Killick-Kendrick R. (1999). The biology and control of phlebotomine sand flies. Clin. Dermatol..

[bib41] Kirstein F., Gray J.S. (1996). A molecular marker for the identification of the zoonotic reservoirs of *Lyme borreliosis* by analysis of the blood meal in its European vector *Ixodes ricinus*. Appl. Environ. Microbiol..

[bib42] Kolářová I., Košťálová T., Talmi-Frank D., Kassahun A., Maia C., Votýpka J., Hailu A., Warburg A., Volf P., Baneth G. (2012). Antibody Response of Domestic Animals to P*hlebotomus Orientalis* Bites.

[bib43] Kostich D.Y. (1951). Source determination of blood meal in sand flies (Phlebotominae) in Yugoslavia (Dobricky County). 2,760 haemoprecipitin tests. Acta Trop..

[bib44] Lainson R., Rangel E.F. (2005). *Lutzomyia longipalpis* and the eco-epidemiology of American visceral leishmaniasis, with particular reference to Brazil: a review. Mem. Inst. Oswaldo Cruz.

[bib45] Lane R.P., Fritz G. (1986). The differentiation of the leishmaniasis *Phlebotomus papatasi* from the suspected *P. bergeroti* (Diptera: phlebotominae). Syst. Entomol..

[bib46] Lewis D.J. (1982). A taxonomic review of the genus *Phlebotomus* (Diptera: Psychodidae). Bull. Br. Mus. Nat. Hist. (Entomol. Ser.).

[bib47] Lutomiah J., Omondi D., Masiga D., Mutai C., Mireji P.O., Ongus J., Linthicum K.J., Sang R. (2014). Blood meal analysis and virus detection in blood-fed mosquitoes collected during the 2006-2007 rift valley fever outbreak in Kenya. Vector Borne Zoonotic Dis..

[bib48] Macedo-Silva V.P., Martins D.R.A., De Queiroz P.V.S., Pinheiro M.P.G., Freire C.C.M., Queiroz J.W., Dupnik K.M., Pearson R.D., Wilson M.E., Jeronimo S.M.B., Ximenes M.D.F.F.M. (2014). Feeding preferences of *Lutzomyia longipalpis* (Diptera: Psychodidae), the sand fly vector, for *Leishmania infantum* (Kinetoplastida: trypanosomatidae). J. Med. Entomol..

[bib49] Maleki-Ravasan N., Oshaghi M.A., Javadian E., Rassi Y., Sadraei J., Mohtarami F. (2009). Blood meal identification in field-captured sand flies: comparison of PCR-RFLP and ELISAAssays. Iran. J. Arthropod-Borne Dis..

[bib50] Miller E., Huppert A. (2013). The effects of host diversity on vector-borne disease: the conditions under which diversity will amplify or dilute the disease risk. PLoS One.

[bib51] Montoya-Lerma J., Lane R.P. (1996). Factors affecting host preference of *Lutzomyia evansi* (Diptera: Psychodidae), a vector of visceral leishmaniasis in Colombia. Bull. Entomol. Res..

[bib52] Morrison A.C., Ferro C., Tesh R.B. (1993). Host preferences of the sand fly *Lutzomyia longipalpis* at an endemic focus of American visceral leishmaniasis in Colombia. Am. J. Trop. Med. Hyg..

[bib53] Morsy T.A., Aboul Ela R.G., Sarwat M.A., Arafa M.A., el Gozamy B.M. (1993). Some aspects of *Phlebotomus papatasi* (scopoli) in greater cairo, Egypt. J. Egypt. Soc. Parasitol..

[bib54] Mukhtar M.M., Sharief A.H., el Saffi S.H., Harith A.E., Higazzi T.B., Adam A.M., Abdalla H.S. (2000). Detection of antibodies of *Leishmania donovani* in animals in Kala azar endemic regions in eastern Sudan: preliminary report. Trans. R. Soc. Trop. Med. Hyg..

[bib55] Muturi C.N., Ouma J.O., Malele I., Ngure R.M., Rutto J.J. (2011). Tracking the feeding patterns of tsetse flies (*Glossina* genus) by analysis of blood meals using mitochondrial cytochromes genes. PLoS One.

[bib56] Nery L.C., Lorosa N.E., Franco A.M. (2004). Feeding preference of the sand flies *Lutzomyia umbratilis* and *L. spathotrichia* (Diptera:Psychodidae, Phlebotominae) in an urban forest patch in the city of Manaus, Amazonas, Brazil. Mem. Inst. Oswaldo Cruz.

[bib57] Ngo K.A., Kramer L.D. (2003). Identification of mosquito blood meals using polymerase chain reaction (PCR) with order-specific primers. J. Med. Entomol..

[bib58] Ngumbi P.M., Lawyer P.G., Johnson R.N., Kiilu G., Asiago C. (1992). Identification of sand fly blood meals from Baringo district, Kenya by direct-enzyme-linked immunosorbent assay (ELISA). Med. Vet. Entomol..

[bib15] Office of the Census Commission and the Central Statistical Agency of Ethiopia (2008). Summary and Statistical Report of the 2007 Population and Housing Census.

[bib59] Oshaghi M.A., Ravasan N.M., Javadian E.A., Mohebali M., Hajjaran H., Zare Z., Mohtarami F., Rassi Y. (2009). Vector incrimination of sand flies in the most important visceral leishmaniasis focus in Iran. Am. J. Trop. Med. Hyg..

[bib60] Palit A., Bhattacharya S.K., Kundu S.N. (2005). Host preference of *Phlebotomus argentipes* and *Phlebotomus papatasi* in different biotopes of West Bengal, India. Int. J. Environ. Health Res..

[bib61] Polanska N., Rohousova I., Volf P. (2014). The role of *Sergentomyia schwetzi* in epidemiology of visceral leishmaniasis in Ethiopia. Parasites Vectors.

[bib62] Quate L.W. (1964). *Phlebotomus* sand flies of the paloich area in the Sudan (Diptera, Psychodidae). J. Med. Entomol..

[bib63] Quinnell R.J., Dye C., Shaw J.J. (1992). Host preferences of the phlebotomine sand fly *Lutzomyia longipalpis* in Amazonian Brazil. Med. Vet. Entomol..

[bib71] R Development Core Team (2012). R: A Language and Environment for Statistical Computing.

[bib64] Rohousova I., Talmi-Frank D., Kostalova T., Polanska N., Lestinova T., Kassahun A., Yasur-Landau D., Maia C., King R., Votypka J., Jaffe C.L., Warburg A., Hailu A., Volf P., Baneth G. (2015). Exposure to *Leishmania* spp. and sand flies in domestic animals in northwestern Ethiopia. Parasites Vectors.

[bib65] Sadlova J., Dvorak V., Seblova V., Warburg A., Votypka J., Volf P. (2013). *Sergentomyia schwetzi* is not a competent vector for *Leishmania donovani* and other *Leishmania* species pathogenic to humans. Parasites Vectors.

[bib66] Saul A. (2003). Zooprophylaxis or zoopotentiation: the outcome of introducing animals on vector transmission is highly dependent on the mosquito mortality while searching. Malar. J..

[bib67] Scott M.C., Harmon J.R., Tsao J.I., Jones C.J., Hickling G.J. (2012). Reverse line blot probe design and polymerase chain reaction optimization for bloodmeal analysis of ticks from the eastern United States. J. Med. Entomol..

[bib68] Soares V.Y.R., da Silva J.C., da Silva K.R., do Socorro Pires e Cruz M., Santos M.P.D., Ribolla P.E.M., Alonso D.P., Coelho L.F.L., Costa D.L., Costa C.H.N. (2014). Identification of blood meal sources of *Lutzomyia longipalpis* using polymerase chain reaction-restriction fragment length polymorphism analysis of the cytochrome B gene. Mem. Inst. Oswaldo Cruz.

[bib69] Srinivasan R., Panicker K.N. (1992). Identification of blood meals of phlebotomine sand flies using the agarose gel diffusion method. Southeast Asian J. Trop. Med. Publ. Health.

[bib70] Svobodova M., Sádlová J., Chang K.P., Volf P. (2003). Short report: distribution and feeding preference of the sand flies *Phlebotomus sergenti* and *P. papatasi* in a cutaneous leishmaniasis focus in Sanliurfa, Turkey. Am. J. Trop. Med. Hyg..

[bib72] Tirados I., Gibson G., Young S., Torr S.J. (2011). Are herders protected by their herds? An experimental analysis of zooprophylaxis against the malaria vector *Anopheles arabiensis*. Malar. J..

[bib73] Torr S.J., Prior A., Wilson P.J., Schofield S. (2007). Is there safety in numbers? The effect of cattle herding on biting risk from tsetse flies. Med. Vet. Entomol..

[bib74] Truppel J.H., Otomura F., Teodoro U., Massafera R., da Costa-Ribeiro M.C.V., Catarino C.M., Dalagrana L., Costa Ferreira M.E.M., Thomaz-Soccol V. (2014). Can equids be a reservoir of *Leishmania braziliensis* in endemic areas?. PLoS One.

[bib75] Valinsky L., Ettinger G., Bar-Gal G.K., Orshan L. (2014). Molecular identification of blood meals from sand flies and mosquitoes collected in Israel. J. Med. Entomol..

[bib76] World Health Organization (March 2010). Control of the leishmaniases: WHO TRS N°949 report of a meeting of the WHO expert committee on the control of leishmaniases, Geneva. WHO Tech. Rep. Ser..

[bib77] Yaghoubi-Ershadi M.R., Javadian E., Kannani A. (1995). Host preference pattern of phlebotomine sand flies of borkhar rural district, Isfahan province, Iran. Acta Trop..

[bib78] Yared S., Deribe K., Gebreselassie A., Lemma W., Akililu E., Kirstein O.D., Balkew M., Warburg A., Gebre-Michael T., Hailu A. (2014). Risk factors of visceral leishmaniasis: a case control study in north-western Ethiopia. Parasites Vectors.

[bib79] Yared S., Gebresilassie A., Akililu E., Balkew M., Warburg A., Hailu A., Gebre-Michael T. (2017). Habitat preference and seasonal dynamics of *Phlebotomus orientalis* in urban and semi-urban areas of kala-azar endemic district of Kafta Humera, northwest Ethiopia. Acta Trop..

